# Integrating Remote Sensing and GIS for Prediction of Winter Wheat (*Triticum aestivum*) Protein Contents in Linfen (Shanxi), China

**DOI:** 10.1371/journal.pone.0080989

**Published:** 2014-01-03

**Authors:** Mei-chen Feng, Lu-jie Xiao, Mei-jun Zhang, Wu-de Yang, Guang-wei Ding

**Affiliations:** 1 Institute of Dryland Farming Engineer, Shanxi Agricultural University, Taigu, People's Republic of China; 2 Department of Chemistry, Northern State University, Aberdeen, South Dakota, United States of America; National Rice Research Center, United States of America

## Abstract

In this study, relationships between normalized difference vegetation index (NDVI) and plant (winter wheat) nitrogen content (PNC) and between PNC and grain protein content (GPC) were investigated using multi-temporal moderate-resolution imaging spectroradiometer (MODIS) data at the different stages of winter wheat in Linfen (Shanxi, P. R. China). The anticipating model for GPC of winter wheat was also established by the approach of NDVI at the different stages of winter wheat. The results showed that the spectrum models of PNC passed F test. The NDVI_4.14_ regression effect of PNC model of irrigated winter wheat was the best, and that in dry land was NDVI_4.30_. The PNC of irrigated and dry land winter wheat were significantly (P<0.01) and positively correlated to GPC. Both of protein spectral anticipating model of irrigated and dry land winter wheat passed a significance test (P<0.01). Multiple anticipating models (MAM) were established by NDVI from two periods of irrigated and dry land winter wheat and PNC to link GPC anticipating model. The coefficient of determination R^2^ (R) of MAM was greater than that of the other two single-factor models. The relative root mean square error (R_RMSE_) and relative error (RE) of MAM were lower than those of the other two single-factor models. Therefore, test effects of multiple proteins anticipating model were better than those of single-factor models. The application of multiple anticipating models for predication of protein content (PC) of irrigated and dry land winter wheat was more accurate and reliable. The regionalization analysis of GPC was performed using inverse distance weighted function of GIS, which is likely to provide the scientific basis for the reasonable winter wheat planting in Linfen city, China.

## Introduction

The winter wheat grain protein content is one of the important standards to evaluate wheat quality [1], [2]. However, the traditional grain protein content (GPC) detection methods need to be destructive in sampling, and consuming time, labor, and money [3]. It is difficult to achieve the quality monitoring and forecasting GPC of the large area of winter wheat. With the rapid development of remote sensing technique in recent years [4], the large scale quickly and nondestructive testing approaches become possible to monitor the wheat grain quality [5], [6], [7].

It is fundamental to realize quantitative remote sensing and precise monitoring methods by building the models of wheat GPC and remote sensing parameters. Apan et al. [8] built the winter wheat GPC estimation model based on spectral vegetation indices by using partial least squares regression method. The model could more accurately predict winter wheat GPC in Australia and the prediction accuracy was 92%. Reyniers et al. [9] monitored the winter wheat GPC using the aerial images and field spectrometer, and the prediction accuracy had achieved 90%. Liu et al. [10] determined the winter wheat GPC using multi-temporal EnviSat-ASAR and Landsat TM satellite images. The model was built based on the C-HH backscatter and SIPI data and the correlation coefficient was 0.75.

Wright et al. [11] analyzed the nitrogen status of wheat plants and found that nitrogen content of flag leaf could predict GPC at middle growth stage in Minidoka County, Idaho. Huang et al. [12] reported that the GPC could be predicted using nitrogen reflectance index and foliar nitrogen concentration around the anthesis stage. The leaf nitrogen content of winter wheat was significantly (P<0.01) correlated with GPC, and spectral vegetation indices significantly correlated to leaf nitrogen content at anthesis stage. Therefore, it was feasible by using remote sensing data to predict GPC at anthesis stage of winter wheat [13].

The normalized difference vegetation index (NDVI) is a most commonly employed vegetation index, which is sensitive to vegetation growth status, productivity, and other biophysical and biochemistry characteristics [14]. It is widely used in the land use cover monitoring [15], vegetation coverage density evaluation [16], crop recognition [17], and crop yield forecast [18], [19] and so on. Stone et al. [20] found that the NDVI and wheat plant nitrogen content were highly correlative at several different growth stages. Freeman et al. [21] demonstrated that NDVI was well correlated with straw N uptake and total N uptake at Feekes growth stages 9 and 10.5 in both cropping cycles at Hennessey and Stillwater.

As there were very limited studies in using vegetation index to simulate the models and predict winter wheat GPC in Shanxi, the current research attempted to predict the GPC of winter wheat in Linfen using plant nitrogen content extracted from satellite remote sensing data. Compared to other studies in the literature [8]–[11], [20], [21], our current research not only established the single-factor model, but also established multiple-factor anticipating model to predict the winter wheat protein contents (different stages) in the identified area. This study also mapped the regionalization characterization of the winter wheat production in the Linfen region based on the GPC character. In addition, the selection of winter wheat variety and management system (*i.e.*, irrigated or dry land) might also lead to different results and need further consideration. The specific objectives were to: (i) extract the planted area and NDVI of different irrigated type winter wheat, (ii) analyze the relation between PNC and NDVI, PNC and GPC, and (iii) build GPC prediction model and realize the regionalization analysis of winter wheat GPC based GIS.

## Materials and Methods

### Site description

**Figure 1:**
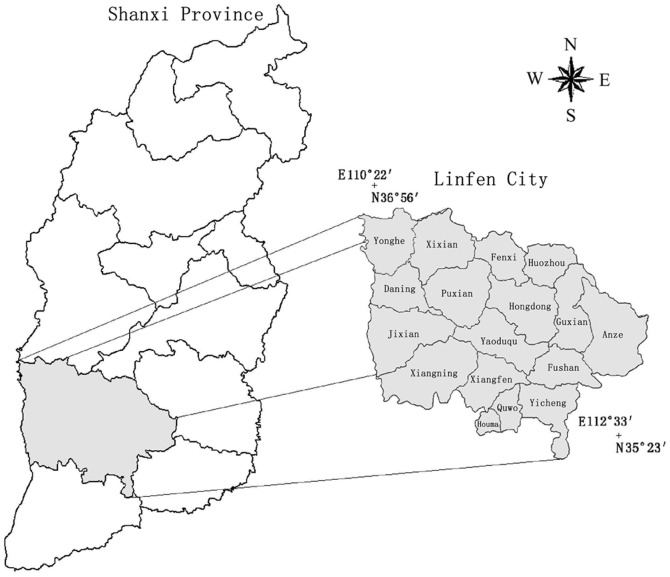
The locations of the study area.

The study was carried out in the southwest of Shanxi province in China (north latitude 35°23′–36°56′, east longitude 110°23′–112°33′) (Figure 1). This region is situated in Loess Plateau and the middle-downstream of Fen River. It is temperate continental monsoon climate in the region. The winter is frigid and dry and the summer is torrid and rainy. The temperature difference between winter and summer is large, and the precipitation is concentrated in summer (June, July, and August). The mean annual temperature is 10.7°C. The average temperature of January and July is −4°C and 26°C, respectively. Frostless season is 180 days annually. The mean annual precipitation is 550 mm. The primary crops include winter wheat, cotton, mealie, and paddy rice. The winter wheat is primarily distributed in central basin where is one of the key winter wheat production areas in Shanxi province, China.

### Sampling and data collection

**Figure 2:**
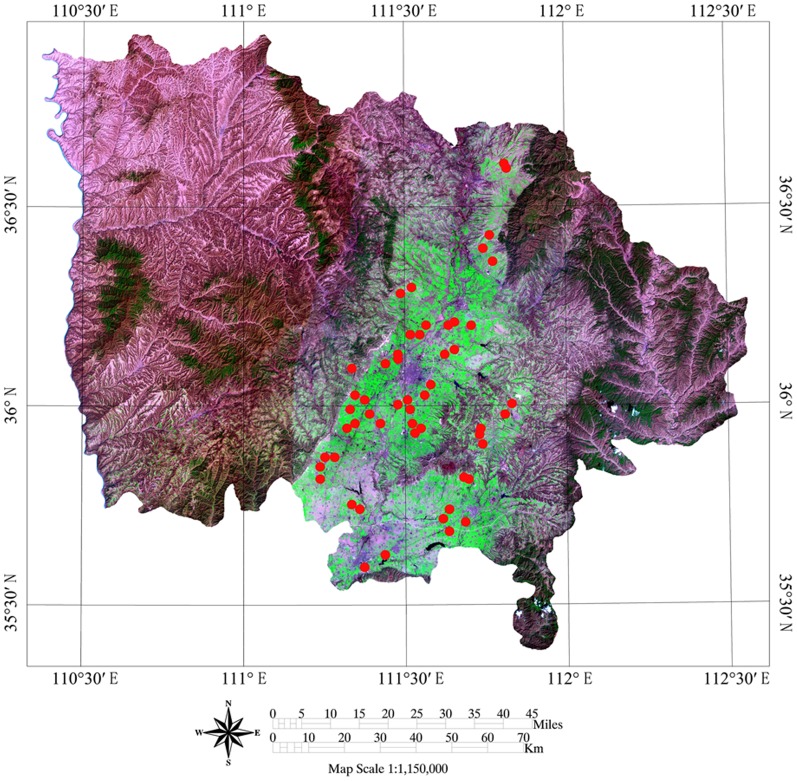
The spatial distribution image of sampling sites.

There were 38 irrigated wheat and 16 dry land winter wheat fields selected from the study areas (Figure 2) in 2006, 2007, and 2009 under the same sowing, fertilization, with a size of at least 5 ha. The irrigated winter wheat main variety was “Linyou 7287,” and the average GPC was 14.06%. The dry land winter wheat main variety was “Jinmai 79,” and the average GPC was 14.84%. Winter wheat both in irrigated and dry land was sowed in early October and harvested in late May (dry land) and mid-June (irrigated wheat) respectively. At the different growth stage (returning green, joining, booting, heading, filling, and maturing stages) of winter wheat, the wheat plant samples were collected in the selected area and threshed manually for plant nitrogen content analysis. At the harvest of wheat, the grains were collected for GPC content determination. More specifically, in each plot, an area of 1 m×1 m was cropped manually just above ground and brought to the lab for processing. For each field, there were triplicate plots (50 m in length by 50 m in width). The GPS was used in the region for accurate positioning. The Aridisols is the predominant soil type in this area. The data collected in 2006 and 2007 were used for model establishment and the data of 2009 were collected to test the established models. We need to note that the field study was authorized by the Agricultural Bureau of Linfen City in Shanxi Province (P. R. China). In addition, no specific permissions were required for these locations/activities because the research activities were for the local agricultural service. Furthermore, the field studies did not involve endangered or protected species and this study also did not involve vertebrate species.

All the samples were placed in the oven to dry at 70°C for 24 h. The PNC (%) and GPC (%) of all the samples were determined by using the Kjeldahl-nitrogen method [22]. The PC in grain was calculated as Kjeldahl-nitrogen content multiplied by 5.7 [23].

Table 1 listed the mean, standard deviation, and range of PNC and GPC values of different irrigation type winter wheat. The range of variation was wide within the wheat variables, *i.e.*, 5-fold and 1.5-fold variation in PNC and GPC of irrigated wheat, 2-fold variation in PNC and GPC of dry land wheat. However, the mean values of different irrigation type wheat were close. And the range of GPC in dry land wheat was so wide; it could be caused by uneven regional rainfall. The wide range of wheat variables was more realistic and universal while the relationship between PNC, GPC and reflectance was made.

**Table 1:** The summary of variables measured different irrigation type winter wheat.

Irrigation Type	Variables	Mean	Standard deviation	Min	Max	Range
irrigated wheat	PNC	1.6690	0.7124	0.7643	3.8479	3.0836
	GPC	12.7617	1.1722	10.4801	15.4102	4.9301
dry land wheat	PNC	1.1928	0.3059	0.8748	1.8144	0.9396
	GPC	12.4966	2.4967	7.1484	14.9925	7.8441

### Data acquisition and treatment of remote sensing data

The remote sensing data in the current study was Landsat TM5 data and MODIS land surface Reflectance (LSR) data synthesized in eight days. The MODIS data to be used were daily level 3 surface reflectance. We downloaded all tiles of h26v05 and h27v05 from the NASA's EOS data gateway, covering the entire extent of Linfen region. Both MODIS and TM data were used in current investigation mainly based on the fact that compared to the TM, MODIS had a higher time resolution and therefore available MODIS was used in time series analysis; and TM had a higher spatial resolution compared to the MODIS, TM could be used for extraction of crop area. Thus, the crop character through a combination of the two datasets could be more effectively monitored.

### TM data preprocessing

Different plants have different seasonal rhythm [17]. The winter wheat growth area was extracted by using TM data (mosaicking disposal has been conducted on purchase) on April 8^th^, 2007 by the Themaic Mapper sensor. The blue (450–520 nm), green (520–600 nm), red (630–690 nm), and near infrared (NIR, 760–900 nm) bands of electromagnetic spectrum were identified at a resolution of 30 m. The planting status of winter wheat in study areas had been identified as the same in recent years and the change of planting area and position could be neglected. Only one scene TM image in 2007 was used to extract planting area, which was conformable with the practical investigation result in 2006.

Preprocessing consisted of:

1. Atmosphere adjustment. The atmospheric correction module FLAASH (Fast Line-of-sight Atmospheric Analysis of Spectral Hypercubes) was used here to adjust the TM remote sensing image [24].
2. Geometric correction. Coarse geometric correction and precise geometric correction were completed using 1∶10 000 digitized raster map and ground control points. The cubic convolution interpolation was used in the georeferencing process to assure that the error was less than one pixel [25].
3. Extraction of researching region. The Mahalanobis Distance taxonomy [26] was used for classification and the best classification effects were reached at the threshold of 2.9. For a fraction of leakage and spillage image after classification, the planting area vectograph produced by classification was superimposed by TM remote sensing image in GIS through second visual interpretation to produce area vectograph ultimately. Then the mask was made and TM image was cropped, thereby the winter wheat planting area was obtained.
4. NDVI calculation. The NDVI of TM image was calculated using the following method [27]:
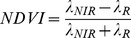
Where 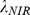 is the reflectance (%) of the near infrared (NIR) band and 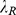 is the reflectance (%) of the red band.

### MODIS data processing

The MODIS data were superior to the TM (in time series resolution identification), NOAA/AVHRR [28] in monitoring of crop growth by features such as high time resolution, high spectral resolution as well as moderate spatial resolution. The MODIS LSR data including two bands (Band1 as red band, 620–670 nm; Band2 as NIR band, 841–876 nm) were obtained from LPDAAC and synthesized in eight days at a 250×250 m spatial resolution. And duration of the data collection was from January to July of 2006, 2007, and 2009 in this study.

Processing consisted of:

1. Image mosaicism. The Mosaicking method [29] localized by geographical coordinates and the Feathering function [30] was used for edge feathering.
2. Geometric correction. The geographical coordinates locating information carried by the head file of MODIS data were used for geometric correction.
3. Atmosphere adjustment. The histogram method [4] was used for the atmosphere adjustment of the images.
4. Extraction of researching region and NDVI calculation. The detailed methods were of the same with TM image.

### Flow scheme of winter wheat planted area extraction

As winter wheat has different growing process under different irrigated conditions, in order to improve the monitoring precision, the irrigated and dry land winter wheat should be distinguished in the study of crop quality and growth monitoring. In general, the winter wheat varieties in irrigated and dry land area of Shanxi province were different and the sowing time was almost the same. However, winter wheat of dry land harvested earlier than of the irrigated mainly because of the longer filling time and low levels of drought stress of irrigated winter wheat.

**Figure 3:**
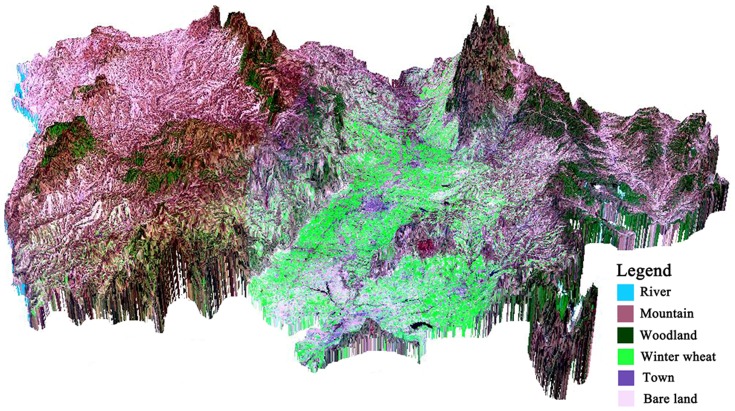
The 3D TM image of remote sensing of Linfen City.

The winter wheat plant area of Linfen region included the irrigated winter wheat area of Linfen basin and dry land winter wheat area of Jinnan hilly region. The irrigated winter wheat area of Linfen basin included hirakawa counties of Houzhou, Hongtong, Linfen, Xiangfen, Quwo, and Houma. The altitude was from 350 to 600 m. The dry land winter wheat area of Jinnan hilly region included most of wheat fields of Fushan and Yicheng counties. The altitude (above sea level) was from 475 to 700 m. The slope of Linfen basin was less than 15°, and the slope of the hilly region was in the range of 15°∼20°. The 3D model image was shown in Figure 3.

We need to clarify the following: (1). Figure 1 was indeed very difficult to distinguish between basins, but 3D remote sensing image in Figure 3 made it clear that the basin was located, *e.g.*, two mountains between L-type, with 20–25 km wide, 200 km long. The irrigated wheat was concentrated in 350–600 m above sea level, dry land was concentrated in 475–700 m above sea level; partial dry land were included in the irrigated wheat elevations. (2). To extract the 3D remote sensing images, by using the ArcScene software, it was hard to figure the superscript in the elevation data. (3). Winter wheat growing region in Linfen could be categorized to two areas including basin area of irrigated winter wheat and in hilly area of dry land winter wheat growing. Two major irrigation systems (river irrigation and well irrigation) managed by local farmers were characterized.

**Figure 4:**
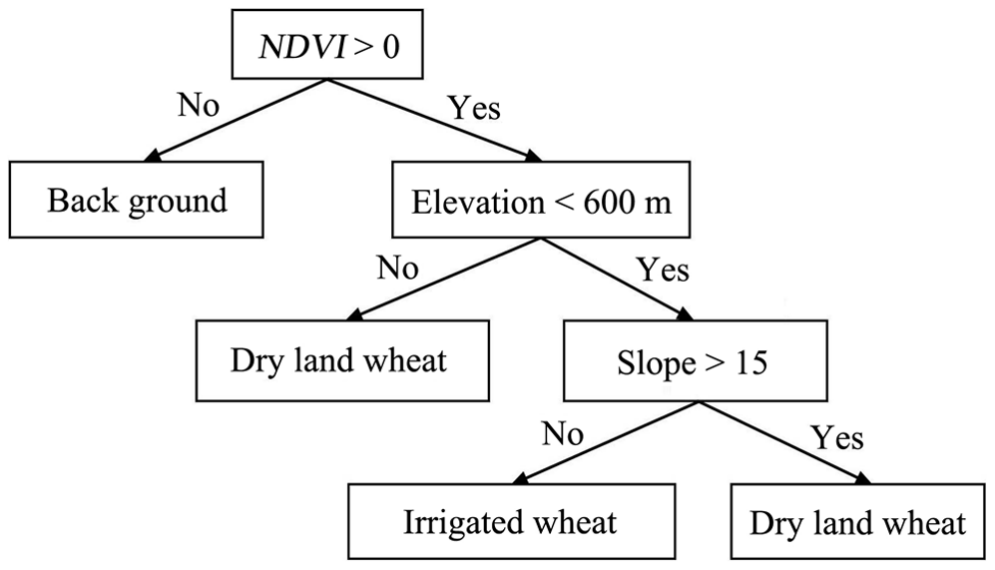
The classification image of decision tree of irrigated and dry land winter wheat.

Using the above-mentioned discrimination characteristics, irrigated and dry land winter wheat plant area were extracted by building the decision tree (Figure 4) extraction model. The NDVI, elevation, and slope values were obtained from TM image of winter wheat planted area and 3D model, respectively. The decision tree structure image was shown in Figure 4 and Table 2 listed the classification results of irrigated and dry land winter wheat. The actual area came from statistical data of agricultural statistics department of Shanxi Province, China. The accuracy estimation was determined by the following formula:
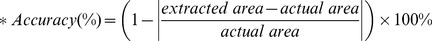



**Table 2:** The classification result of winter wheat in Linfen City.

Winter wheat	Pixel numbers	Extracting area/ha	Actual area/ha	Accuracy/%
Irrigated wheat	1194314	107488	94400	86.15
Dry land wheat	1414335	127290	147733	86.16
Total area	2608649	234778	242133	96.96

### Data analysis and calculation methods

Excel software was used for data collation, analysis, and mapping. All data were analyzed statistically to use DPS (i.e., statistical analysis, regression analysis, and variance analysis). The DPS (Data Processing System) 7.05 is a kind of statistical analysis software [31].

The simple linear regression models were established using the data of PNC and NDVI values and PNC and GPC. Two prediction models were combined into GPC monitoring models. The GPC model was grain protein content monitoring model. Its direct variable was PNC, and indirect variable was NDVI.

The assessment model was based on multiple correlation coefficients [32], F-test values of significance [7], relative root mean square errors (*R_RMSE_*) [33], and relative error (*RE*) [34].

The relative root mean square error (*R_RMSE_*) and relative error (*RE*) are defined by:
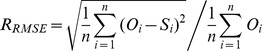


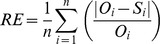
Where, 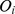 and 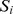 are the observed and simulated values of the sample i and n is the number of samples.

Sampling points monitored by current study were conducted in the state of nature. The research process did not interfere with the farmer's production and operation, in this case the coefficient of determination of the model, which can meet the needs of the model.
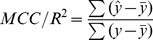


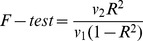
Where 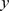, 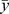 and 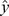 are the observed value, mean value, and prediction value, degrees of freedom 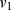 = m, 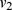 = n-m-1.

## Results and Discussion

### Relation of NDVI and nitrogen content of plants

**Figure 5:**
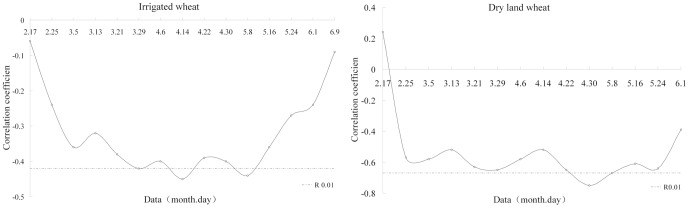
Correlation coefficients between NDVI and plant nitrogen content of irrigated and dry land wheat at different stages.

The correlation between NDVI and nitrogen content of plants was established. The degree of correlation was different for different irrigation type winter wheat at different growth stages (Figure 5). Through data analysis, there was a significant (P<0.01) negative correlation between NDVI_4.14_, NDVI_5.8_ and nitrogen content of plants of irrigated winter wheat, *i.e.*, at early heading stage (May 8) the correlation coefficient were −0.45 and −0.44, respectively. There was a significant difference (P<0.01) between NDVI_4.30_, NDVI_5.8,_ and nitrogen content of plants of dry land winter wheat. The max-relativity also appeared in the May (8^th^) and the correlation coefficient were −0.75 and −0.67, respectively. Our data might suggest that the NDVI could be used as the indicators to predict nitrogen content of irrigated and dry land winter wheat plants in Shanxi.

According to the relationship between NDVI and nitrogen content of different irrigation type winter wheat plants, the statistical evaluation models of nitrogen content of plants were developed. The simulation models were showed in Table 3. The results of the regression models and significant F-tests were conducted for all models (Table 3). The models of NDVI_4.14_ and NDVI_4.30_ had the best prediction effects on irrigated and dry land, respectively. It was clear that all R^2^ of the irrigated wheat models were lower than dry land and the R_RMSE_ and RE were higher than dry land. This result demonstrated that dry land models had a better ability to predict nitrogen content of winter wheat.

**Table 3:** The statistical evaluation models of nitrogen content of different irrigation type winter wheat.

Irrigation Type	Model	R^2^	F–test	F–crit	R_RMSE_	RE
Irrigated wheat	N(%) = 2.7597–0.0241NDVI_4.14_	0.202	9.1	7.4	0.369	0.291
	N(%) = 2.9790–0.0225NDVI_5.8_	0.192	8.6	7.4	0.346	0.278
Dry land wheat	N(%) = 3.4194–0.0440NDVI_4.30_	0.569	18.5	8.5	0.131	0.111
	N(%) = 2.7682–0.0281NDVI_5.8_	0.446	11.3	8.5	0.171	0.146

Theoretically, the R^2^ of model should be closer to 1 for ideal result, and RE, RMSE should be closer to 0 for ideal result. However, in this work, R^2^ was only 0.569 for dry land wheat, but the model passed a 0.01 significant level test. The large sample numbers could be one of the reasons. In addition, we did not interfere with farmers' production and management in the whole study process; this might also be the main reason for the emergence of such results. Therefore, it speculated that there might be a possibility by using remote sensing data for large area monitoring winter wheat protein content.

And the multiple anticipating models were also established using two temporal NDVI and nitrogen content of plant, the models were as follows:

Irrigated winter wheat: 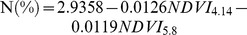, (n = 38, R^2^ = 0.207, F = 9.4, R_RMSE_ = 0.302, RE = 0.254)

Dry land winter wheat: 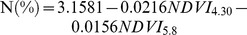, (n = 16, R^2^ = 0.527, F = 15.6, R_RMSE_ = 0.124, RE = 0.106)

The above mentioned multiple anticipating models (MAM) showed that they had passed F test and also reached significant level (P<0.01). The R^2^ value of MAM was higher than the unifactor models for irrigated winter wheat. The dry land R^2^ of MAM value was in the medium between the two unifactor models.

### Relation of nitrogen content of plants and GPC

The correlation between PNC and GPC was carried out at different stages. There was a significant (P<0.01) positive correlation between plant nitrogen content and GPC of irrigated and dry land winter wheat in early heading (the correlation coefficient of 0.541 and 0.567, respectively). The difference was not significant (P>0.01) in other stages. And the agronomy models were established to predict GPC; the models were showed in Table 4.

**Table 4:** The agriculture statistical evaluation models of GPC of different irrigation type winter wheat.

Irrigation Type	Model	R^2^	F–test	F–crit	R_RMSE_	RE
Irrigated wheat	Pro (%) = 10.7421+1.2101N (%)	0.541	42.4	7.4	0.063	0.054
Dry land wheat	Pro (%) = 5.1689+ 6.1431N (%)	0.567	18.3	8.5	0.205	0.141

The F-test value of the GPC evaluation models was greater than F-critical value (Table 4). It indicated that the models passed a 0.01 significant level test. The R_RMSE_ and RE of irrigated and dry land wheat models were 0.063 and 0.054, 0.205 and 0.141, respectively. It showed that the two models were more suitable for prediction.

It was also noted that the former researches for constructing model were mainly concentrating on mixing the different irrigation type winter wheat to monitor the crop growth status and estimate crop yield. In fact, the growing process of irrigated and dry land winter wheat as affected by varieties and environmental factor was of difference. For example, the growth stage for irrigated land winter wheat was middle-heading stage on May 8; while late-heading stage for dry land winter wheat was observed on May 8. In addition, the dry land winter wheat matured on June 1; while for irrigated land wheat was on June 9. Thus, it was hard to construct the mixed model based on the specific growth stage. The models were simulated in our investigation only in dry land wheat and irrigated wheat.

### Spectral GPC estimation models

Because PNC was strongly associated with GPC, the PNC could indirectly be used to predict GPC though the correlation between PNC and vegetation index. The spectral GPC estimation models were established by using the plant nitrogen content as the connecting point as shown in Table 5.

**Table 5:** The spectral GPC estimation models of irrigated and dry land winter wheat.

Irrigation Type	Model	R^2^	F–test	F–crit	R_RMSE_	RE
Irrigated wheat	Pro(%) = 14.0815–0.0292NDVI_4.14_	0.235	11.1	7.4	0.083	0.063
	Pro(%) = 14.3469–0.0272NDVI_5.8_	0.228	10.6	7.4	0.082	0.063
Dry land wheat	Pro(%) = 26.1742–0.2700NDVI_4.30_	0.487	13.3	8.5	0.185	0.123
	Pro(%) = 22.1738–0.1725NDVI_5.8_	0.557	17.6	8.5	0.189	0.119

All the F values of spectral prediction models for GPC in irrigated and dry land were greater than the value of F-critical, revealing that all of the prediction models for GPC of irrigated and dry land winter wheat passed the significance level test of 0.01. The R values of the models constructed by NDVI_4.14_ for irrigated winter wheat were higher than those of the models constructed by NDVI_5.8_, therefore the model in April (14^th^) was selected as the prediction models for GPC of irrigated winter wheat. The model in May (8^th^) was selected as the prediction models for GPC of dry land winter wheat.

Simultaneously, hybrid prediction model was constructed by NDVI in two time phases of irrigated and dry land winter wheat as well as nitrogen content in plants to link the prediction model for GPC, the formulas were as following:

Irrigated winter wheat:


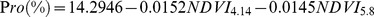
, (n = 38, R^2^ = 0.244, F = 11.6, R_RMSE_ = 0.081, RE = 0.062);

Dry land winter wheat:


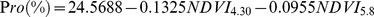
, (n = 16, R^2^ = 0.632, F = 24.0, R_RMSE_ = 0.144, RE = 0.106).

Significance level test of correlation coefficient to the formulas above with F test indicated that spectral GPC prediction models for winter wheat in all regions passed the significance level test of 0.01, which showed highly significant relationships between NDVI and GPC. The purpose of this model construction was just for prediction, and then R^2^ could be used to test the models [35]. The R^2^ values of hybrid prediction models for irrigated and dry land winter wheat were larger than those of the other two unifactorial models. Additionally, R_RMSE_ and RE were smaller than the two unifactorial models, lowered 0.2% (0.1%) and 1.7% (1.3%), respectively.

Furthermore, we constructed a mixed GPC model of irrigated and dry land wheat by using the same growth stage data (8^th^ of May). The formula listed as the following:


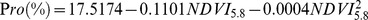
, (n = 54, R^2^ = 0.174, F = 6.57, R_RMSE_ = 0.254, and RE = 0.340)

Although the GPC mixed prediction model for irrigated and dry land winter wheat passed the significance level test of 0.01, the R^2^ value of mixed model was lower than classification models; the R_RMSE_ and RE were higher. Again, the growing process of irrigated land and dry land winter wheat as affected by varieties and environmental factor was of difference. For example, the growth stage for irrigated land winter wheat was middle-heading stage on May 8; while late-heading stage for dry land winter wheat was observed on May 8. In addition, the maturity stage was also different for both irrigated wheat and dry land wheat. Thus, our results demonstrated that the precision of mixed model was far below the classification models using the same stage data.

**Figure 6:**
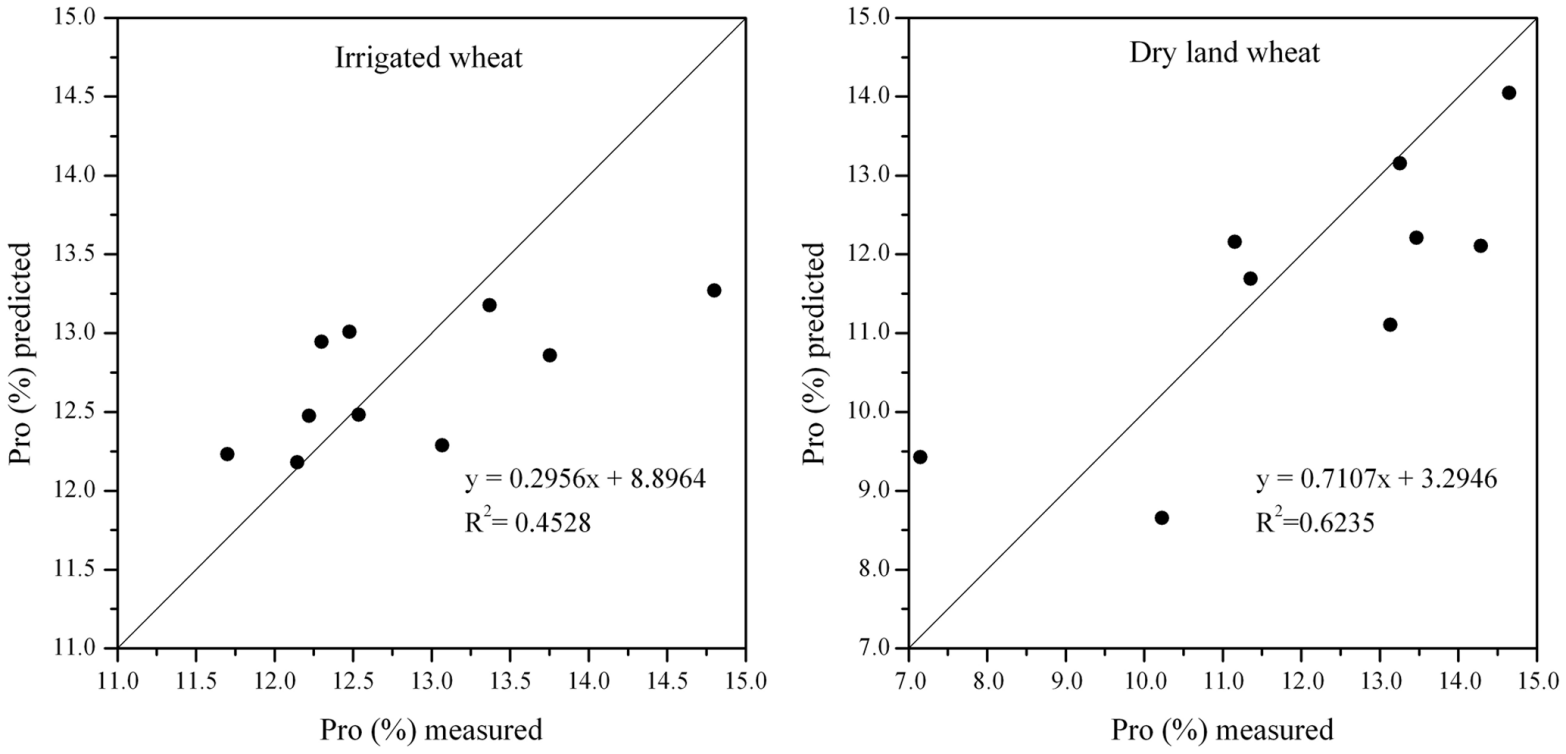
Liner relationships between predicted and measured values of GPC of irrigated and dry land wheat.

Additionally, based on our study, liner relationship between predicted and measured values of GPC (independent dataset from 2009) of irrigated and dry land wheat was further explored in Figure 6. The GPC (%) was predicted from NDVI_4.14_ and NDVI_5.8_, resulting in a prediction of GPC [R^2^ = 0.453, R_RMSE_ = 0.054, slope = 0.296 (p<0.01), and intercept = 8.896] for irrigated winter wheat. The combination of NDVI_4.30_ and NDVI_5.8_ was an excellent predictor of GPC content [R^2^ = 0.624, R_RMSE_ = 0.118, slope = 0.711 (p < 0.01), and intercept = 3.295] for dry land wheat. In sum, the test results of hybrid prediction models were better than those of the unifactorial models and the hybrid prediction models would be more reliable in predicting the GPC of irrigated and dry land winter wheat.

### The regionalization analysis of irrigated and dry land winter wheat

**Figure 7:**
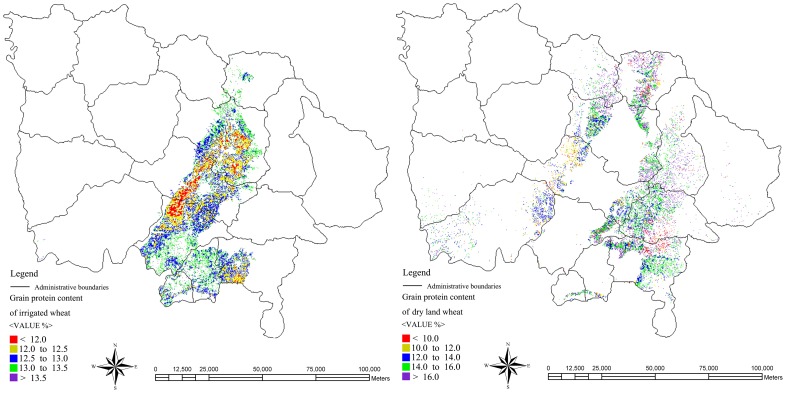
The GPC spatial distribution images of irrigated and dry land wheat.

The spatial distribution maps of irrigated and dry land winter wheat GPC were composited according to the hybrid protein prediction models and images operation (Figure 7). From this graph, it demonstrated that irrigated winter wheat with lower GPC than 12.5% was mainly distributed in cities and counties such as Hongtong, Linfen, Xiangfen, and Yicheng. Winter wheat with GPC between 12.5% and 13% was unequally distributed in all counties. Winter wheat with GPC between 13% and 13.5% was mostly distributed in the North and South region. Winter wheat having higher GPC than 13.5% was scattered distributed in all counties. As for in dry land, winter wheat with GPC between 10% and 12% was mainly distributed in cities and counties such as Linfen, Huozhou, and Xiangning; while winter wheat with GPC between 14% and 16% was primarily distributed in the Southeast region.

**Figure 8:**
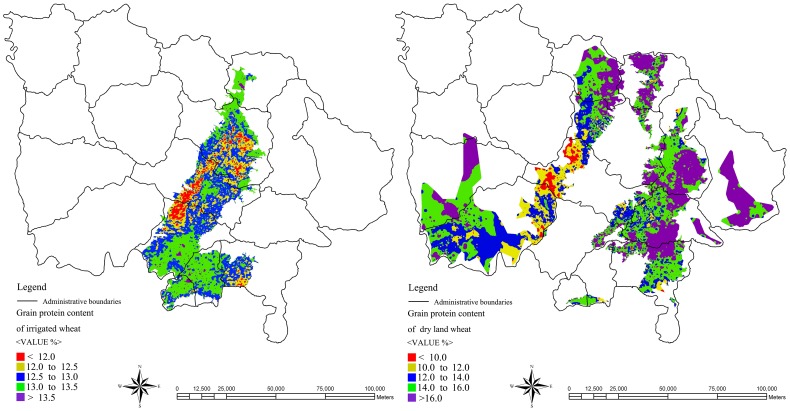
The GPC regionalization images of irrigated and dry land wheat.

From Figure 7, it was noted that the spatial distribution of different winter wheat GPC was comparatively scattered in a large scale and GPC in different point had significant difference. Therefore, it was very difficult to play a key role for winter wheat practice with various GPC. This paper focused on the spatial analysis capability by GIS aiming for better regionalization analysis of winter wheat. The outermost dispersion points were connected to form wheat planting region and the Kriging interpolation method [36] was used to realize wheat GPC regionalization (Figure 8). The special distribution images of winter wheat GPC of irrigated and dry land were introduced into ArcGIS, and translated into point layer. Then the peripheral point outline was drawn to form the polygon layer. The regionalization analysis of GPC was performed using inverse distance weighted function, clipped by the polygon layer. The results were showed in Figure 8.

### Summary

In this study, taking nitrogen content as the connecting point, a prediction model for GPC in winter wheat based on different breeding periods was established. Compared to the other two unifactor models, the values of R^2^ of MAM of winter wheat in irrigated and dry land were greater. Simultaneously, R_RMSE_ and RE in the MAM were lower than those in the unifactor models, lowering 0.1% (0.1%) and 1.7% (1.3%), respectively. Generally, all of the prediction results in the MAM for GPC were better than those in the unifactor models. Therefore, the MAM for forecasting GPC of winter wheat in irrigated and dry land will be more accurate and reliable. And the analysis of regionalization in GPC of irrigated and dry land winter wheat was preceded in ArcGIS, which could serve wheat research and production well.

The mean, min, and max values of the measured GPC (Table 1) of irrigated wheat were all higher than that of the dry land wheat. However, as shown in Figure 7 and 8, the most of the predicted GPC values of irrigated wheat appeared lower than those of the dry land wheat. This weird observation might be explained by the following. On the one hand, the difference mainly came from the modeling precision (e.g., R^2^ = 0.244 for irrigated wheat was much less than R^2^ = 0.632 for dry land wheat). In addition, actual wheat planting area was scattering in the whole dry land wheat distribution region. Therefore, by statistical interpolation statistics method, the dry land wheat planting area was unilateral enlarged.

Most of the previous researchers [2], [23] established the hyperspectral model for nitrogen and protein contents in leaf in the flowering period to forecast GPC of winter wheat. However, in the monitoring of large areas, because of difficulty in selection of sampling point, punctuality of sampling, and storage of samples, nitrogen content of different growth periods was selected for calculation in this article. Consequently, this method needs further study and consolidate the applications of established model.

Ideally, once spectral signatures are available it should be able to predict the satisfactorily the GPC under diverse condition. However, in the large area of winter wheat by remote sensing monitoring study, due to the choice of sample sites (related to transportation, information and communications, science and technology and other factors) and timeliness of the sample for the entire region are difficult to achieve synchronous sampling. Furthermore, the inconsistencies in sampling time and the preservation of the samples are also inevitable. This article focused only on the plant nitrogen content-related research. The forecast accuracy needs to be further study; establishing a research base on the ground may be the key to addressing this problem.

In order to improve the precision of the prediction model and minimize the interference of the mixed pixels, the TM and MODIS data were used in this study through overlay analysis, spatial scaling, adjust MODIS spatial resolution to match TM data. However, due to the terrain surface complexity and the scattered (fragmentation) of the winter wheat sowing area in the whole study region, the mixed pixels interferences were not completely erased and still existed in some degree. Hence, it is necessary to summarize current research to identify (develop) the appropriate technique of partition of mixed pixels for future research.

Meteorological factors including rainfall, temperature, and sunshine have the important influence on quality of grain. The corresponding correlation analysis of meteorological factors was conducted (unpublished data) and the correlation was not significant. The meteorological factors were not brought into establishment of the model in this study. However it did not mean meteorological factors had no effect on grain quality. The indication of insignificant correlation was probably due to distant sampling points to weather stations. The factors such as cultivars (wheat variety), fertilizers [37], [38] also affect grain quality of winter wheat which was not involved in the study. Thereafter, more solid study on the factors affecting quality of winter wheat needs for more consideration. In the end, remote sensing monitoring and regionalizing of GPC of winter wheat was dissected in the study and monitoring and regionalizing of other qualities of winter wheat need further research.

## References

[1] Shewry PR 2004/Rev. (2006) Improving the protein content and quality of temperate cereals: wheat, barley and rye. In Impacts of agriculture on human health and nutrition [online]. [cit. 2010-04-08]. EOLSS website. Available: http://www.eolss.net/ebooks/Sample%20Chapters/C10/E5-21-04-04.pdf. Accessed 2013 November 9.

[2] Matsunak T, Watanabe Y, Miyawaki T, Ichikawa N (1997) Prediction of grain protein content in winter wheat through leaf color measurements using a chlorophyll meter. Journal of Soil Science & Plant Nutrition 43 (1) 127–134.

[3] Wang ZJ, Wang JH, Liu LY, Huang WJ, Zhao CJ, et al (2004) Prediction of grain protein content in winter wheat (Triticum aestivum L.) using plant pigment ratio (PPR). Field Crops Research 90: 311–321.

[4] Lu D, Mausel P, Brondizio E, Moran E (2004) Change detection techniques. International Journal of Remote Sensing 25: 2365–2407.

[5] Gitelson AA, Kaufman YJ (1998) MODIS NDVI optimization to fit the AVHRR data series-Spectral considerations. Remote Sensing of Environment 66: 343–350.

[6] Hansen PM, Jorgensen JR, Thomas A (2002) Predicting grain yield and protein content in winter wheat and spring barley using repeated canopy reflectance measurements and partial least squares regression. Journal of Agricultural Science 139: 307–318.

[7] Manjunath KR, Potdar MB (2002) Large area operational wheat yield model development and validation based on spectral and meteorological data. International Journal of Remote Sensing 23 (15) 2023–3038.

[8] Apan A, Kelly RW, Phinn SR, Strong WM, Lester BD, et al (2006) Predicting grain protein content in wheat using hyperspectral sensing of in-season crop canopies and partial least squares regression. International Journal of Geoinformatics 2 (1) 93–108.

[9] Reyniers M, Vrindts E, Baerdemaeker JD (2006) Comparison of an aerial-based system and an on the ground continuous measuring device to predict yield of winter wheat. European Journal of Agronomy 24 (2) 87–94.

[10] Liu LY, Wang JJ, Bao YS, Huang WJ, Ma ZH, et al (2006) Predicting winter wheat condition, grain yield and protein content using multi-temporal EnviSat-ASAR and Landsat TM satellite images. International Journal of Remote Sensing 27 (4) 737–753.

[11] Wright DL, Rasmussen VP, Ramsey RD, Ellsworth JW (2004) Canopy reflectance estimation of wheat nitrogen content for grain protein management. GIScience and Remote Sensing 41 (4) 287–300.

[12] Huang WJ, Wang JH, Song XY, Zhao CJ, Liu LY (2007) Wheat grain quality forecasting by canopy reflected spectrum. International Federation for Information Processing. Volume 259: Computer And Computing Technologies In Agriculture, Volume II: 1299–1301.

[13] Zhao CJ, Liu LY, Wang JH, Huang WJ, Song XY, et al (2005) Predicting grain protein content of winter wheat using remote sensing data based on nitrogen status and water stress,. International Journal of Applied Earth Observation and Geoinformation 7 (1) 1–9.

[14] Boken VK, Shaykewich CF (2002) Improving an operational wheat yield model using phonological phase-based Normalized Difference Vegetation Index. International Journal of Remote Sensing 23 (20) 4155–4168.

[15] Shih SF (1994) NOAA Polar-Orbiting satellite HRPT data and GIS in vegetation index estimation for the everglades agricultural area. Soil and Crop Science Society of Florida Proceedings 53: 19–24.

[16] Zribi M, Hégarat-Mascle L (2003) Derivation of Wild Vegetation Cover Density in Semi-arid Region: ERS2/SAR Evaluation. International Journal of Remote Sensing (24) 1335–1352.

[17] Yafit C, Maxim S (2002) A national knowledge-based crop recognition in Mediterranean environment. International Journal of Applied Earth Observation and Geoinformation 4: 75–87.

[18] Daughtry CST, Gallo KP, Goward SN, Prince SD, Kustas WP (1992) Spectral estimates of absorbed radiation and phytomass production in corn and soybean canopies. Remote Sensing of Environment 39: 141–152.

[19] Serrano L, Filella I, Peñuelas J (2000) Remote sensing of biomass and yield of winter wheat under different nitrogen supplies. Crop Science 40: 723–731.

[20] Stone ML, Solie JB, Raun WR, Whitney RW, Taylor SL, et al (1996) Use of spectral radiance for correcting in-season fertilizer nitrogen deficiencies in winter wheat. Trans ASAE 39: 1623–1631.

[21] Freeman KW, Raun WR, Johnson GV, Mullen RW, Stone ML, et al (2003) Late-season prediction of wheat grain yield and grain protein. Communications in Soil Science Plant Analysis 34 (13) 1837–1852.

[22] Sahrawat KL (1995) Fix ammonium and carbon-nitrogen ratios of some semi-arid tropical Indian soils. Geoderma 68: 219–224.

[23] Reyniers M, Vrindts E (2006) Measuring wheat nitrogen status from space and ground-based platform. International Journal of Remote Sensing 27 (3) 549–567.

[24] Agrawal G (2011) Comparision of QUAC and FLAASH atmospheric correction modules on EO-1 Hyperion data of Sanchi. International Journal of Advanced Engineering Sciences and Technologies 4 (1) 178–186.

[25] Thomlinson JR, Bolstad PV, Cohen WB (1999) Coordinating Methodologies for Scaling Landcover Classifications from Site-Specific to Global. Steps toward Validating Global Map Products 70 (1) 16–28.

[26] Maria-Pza D, Christian C, Borja M, Pilar B, Constantino V, et al (2012) Grapevine yield and leaf area estimation using supervised classification methodology on RGB images taken under field conditions. Sensors 12: 16988–17006. 23235443 10.3390/s121216988 PMC3571822

[27] Rouse JW, Haas RH, Schell JA, Deering DW (1974) Monitoring vegetation systems in the great plains with ERTS//NASASP 2351, third ERTS 21 Symposium, Washington, D. C. 309–317.

[28] Gitelson AA, Kaufman YJ (1998) MODIS NDVI optimization to fit the AVHRR data series-Spectral considerations. Remote Sensing of Environment 66: 343–350.

[29] Shum HY, Szeliski R (2000) Systems and experiment paper: construction of panoramic image mosaics with global and local alignment. International Journal of Computer Vision 36 (2) 101–130.

[30] Peter JB, Edward HA (1983) A multiresolution spline with application to image mosaics. Acm Transactions on Graphies 2 (4) 217–236.

[31] Tang QY, Zhang CX (2013) Data Processing System (DPS) software with experimental design, statistical analysis and data mining developed for use in entomological research. Insect Science 20: 254–260. 23955865 10.1111/j.1744-7917.2012.01519.x

[32] Abdi H (2007) Multiple correlation coefficient. In N.J. Salkind (Ed.): Encyclopedia of Measurement and Statistics. Thousand Oaks (CA): Sage, 648–651.

[33] Marco G, Richard WM, Detlev H (2006) Influence of three-dimensional cloud effects on satellite derived solar irradiance estimation—First approaches to improve the Heliosat method. Solar Energy 80 (9) 1145–1159.

[34] White GC, Anderson DR, Burnham KP, Otis DL (1982) Capture-recapture and removal methods for sampling closed populations. Los Alamos National Laboratory, Los Alamos.

[35] Guiarati DN (1995) Basic econometrics. 3rd edition (New York: McGraw Hill).

[36] Matheron G (1963) Principles of Geostatistics. Economic Geology 58: 1246–1266.

[37] Stark J, Souza E, Brown B, Windes J (2001) Irrigation and Nitrogen Management Systems for enhancing Hard Spring Wheat Protein. American Society of Agronomy Annual Meetings. Charlotte, North Carolina, October 24, 2001. Available: http://www.extension.uidaho.edu/swidaho/nutrient%20management/asa%20protein%20presentatns/2001asastark1/index.htm. Accessed: 9 Nov 2013

[38] Terman GL, Ramig RE, Dreier AF, Olson RA (1969) Yield-protein relationships in wheat grain as affected by nitrogen and water. Agronomy Journal 611: 755–759.

